# *Arquivos de Neuro-Psiquiatria* : 82 years old, a new phase begins now

**DOI:** 10.1055/s-0045-1804506

**Published:** 2025-02-24

**Authors:** Hélio Afonso Ghizoni Teive, Ayrton Roberto Massaro, Carlos Henrique Ferreira Camargo, Clécio de Oliveira Godeiro

**Affiliations:** 1 Universidade Federal do Paraná, Hospital de Clínicas, Departamento de Medicina Interna, Curitiba PR, Brazil; 2 Hospital Sírio-Libanês, São Paulo SP, Brazil; 3 Universidade Federal do Paraná, Hospital de Clínicas, Programa de Pós-Graduação em Medicina Interna, Curitiba PR, Brazil; 4 Universidade Federal do Rio Grande do Norte, Hospital Universitário Onofre Lopes, Departamento de Neurologia, Natal RN, Brazil

In 2025, *Arquivos de Neuro-Psiquiatria* (ANP), the esteemed scientific journal of the Brazilian Academy of Neurology (ABN), will celebrate its 82 ^nd^ year of continuous publication since its inception under the leadership of founding editor Professor Oswaldo Lange. The journal has since benefited from the distinguished contributions of Editor Emeritus Professor Antonio Spina-França Neto and past Editors-in-Chief Professors José Antonio Livramento and Luís dos Ramos Machado. In a new phase for ANP, past Editor-in-Chief Paulo Caramelli along with current Editors-in-Chief Hélio Afonso Ghizoni Teive and Ayrton Roberto Massaro, supported by the ABN, have enacted significant changes to the journal's editorial process, with more developments anticipated in the coming year. 1 2

For several years, the editors-in-chief of ANP have deliberated with the Editorial Board and the ABN on adopting an English-language name for ANP. The traditional name, used since 1945, has contributed significantly to the global recognition of Brazilian neurology. However, despite its historical significance and affectionate regard among ABN members, the name poses two main challenges. Firstly, it correctly references neurology and the ABN but also includes psychiatry, an independent specialty with its own professional societies and scientific publications in Brazil. Although ANP previously published content on psychiatry, more recently, submissions focused solely on psychiatric themes will no longer be accepted. Secondly, since ANP has transitioned to accepting submissions exclusively in English, there has been a notable increase each year in the diversity of authors from various countries across all continents. Consequently, the full internationalization of the journal, necessitating English-only article titles and abstracts, and the journal title, has become essential to enhance and maintain ANP's visibility and prominence on a global scale. This transition has been a meticulous and slow bureaucratic process; however, we have now reached a consensus with regulatory agencies to adopt a new name. Starting this year, ANP will be known as “ *Arquivos de Neuro-Psiquiatria:* *Neurology and Neuroscience* ”.

In 2025, the journal will also see updates in the roles of Associate Editors and the introduction of new sections for article submission and publication. The “Practice Neurology” section aims to attract new researchers, including residents and postgraduate students, by accepting manuscripts that comprehensively discuss diagnosis and treatment through clinical cases or case series. This section is designed to foster a rich learning environment and facilitate sharing experiences. The “Point of View” section will address controversial topics divided into three parts: pro, con, and an analysis synthesizing the arguments presented. This section will feature contributions from experts specializing in the issues under debate.

These enhancements aim to elevate the journal's scientific quality, increasing its reach not only in Latin America, where it is already a leading publication in neurology, but also globally. We anticipate these changes will significantly raise the journal's impact factor, reflecting the improved quality of the articles published and reinforcing the stature of the ANP and Brazilian neurology.
